# An Investigation of the Effects of Self-Assembled Monolayers on Protein Crystallisation

**DOI:** 10.3390/ijms140612329

**Published:** 2013-06-07

**Authors:** Chen-Yan Zhang, He-Fang Shen, Qian-Jin Wang, Yun-Zhu Guo, Jin He, Hui-Ling Cao, Yong-Ming Liu, Peng Shang, Da-Chuan Yin

**Affiliations:** 1 Institute for Special Environmental Biophysics, Key Laboratory for Space Bioscience and Biotechnology, School of Life Sciences, Northwestern Polytechnical University, Xi’an 710072, Shaanxi, China; E-Mails: zhangchenyan@nwpu.edu.cn (C.-Y.Z.); shenhefang@mail.nwpu.edu.cn (H.-F.S.); guoyunzhu@mail.nwpu.edu.cn (Y.-Z.G.); hej@mail.nwpu.edu.cn (J.H.); hlcao@mail.nwpu.edu.cn (H.-L.C.); auliuym@mail.nwpu.edu.cn (Y.-M.L.); shangpeng@nwpu.edu.cn (P.S.); 2 Shaanxi Research Design Institute of Petroleum and Chemical Industry, Xi’an 710054, Shaanxi, China; E-Mail: wangqinjin19821982@126.com

**Keywords:** protein crystallisation, self-assembled monolayer, methyl, sulfydryl, amino

## Abstract

Most protein crystallisation begins from heterogeneous nucleation; in practice, crystallisation typically occurs in the presence of a solid surface in the solution. The solid surface provides a nucleation site such that the energy barrier for nucleation is lower on the surface than in the bulk solution. Different types of solid surfaces exhibit different surface energies, and the nucleation barriers depend on the characteristics of the solid surfaces. Therefore, treatment of the solid surface may alter the surface properties to increase the chance to obtain protein crystals. In this paper, we propose a method to modify the glass cover slip using a self-assembled monolayer (SAM) of functional groups (methyl, sulfydryl and amino), and we investigated the effect of each SAM on protein crystallisation. The results indicated that both crystallisation success rate in a reproducibility study, and crystallisation hits in a crystallisation screening study, were increased using the SAMs, among which, the methyl-modified SAM demonstrated the most significant improvement. These results illustrated that directly modifying the crystallisation plates or glass cover slips to create surfaces that favour heterogeneous nucleation can be potentially useful in practical protein crystallisation, and the utilisation of a SAM containing a functional group can be considered a promising technique for the treatment of the surfaces that will directly contact the crystallisation solution.

## 1. Introduction

In recent decades, remarkable advances in promoting protein crystallisation have been achieved [1,2]. Due to the wide application of automation and availability of synchrotron radiation in protein crystallisation, the total number of successfully resolved structures deposited in the PDB database has sharply increased to more than 89,393 until 2 April, 2013 [3]. However, the overall success rate of obtaining diffraction quality crystals from a purified soluble protein remains at a very low level (approximately 15%) [4]. New methods to promote protein crystallisation are still necessary.

Protein crystallisation proceeds in two steps: nucleation and growth [5]. Successful crystallisation depends on the success of both steps. As the first step, nucleation is very important because crystals can never be obtained without nucleation. Generally, there is an energy barrier for the formation of nuclei in the solution [6]. To succeed in crystallisation, the energy barrier must be overcome. There are several methods to lower the energy barrier, among which heterogeneous nucleation is widely accepted as an important approach [7].

Heterogeneous nuclei, from which crystal growth begins, are typically in the form of solid particles in the crystallisation solution or a solid interface in contact with the solution. Solid particles such as gel glass, sephadex beads, carbon powder, alumino-silicate, molecular sieves, zeolite, dried seaweed and horse hair have proven to be effective as the heterogeneous nucleus [8–10]. In most cases, the solid particles must be added to the crystallisation solution. However, no matter how these nuclei are added (automatically or manually), this procedure typically requires an additional step in standard protocols.

Solid surfaces of a relatively larger size (than the small particles) were also tested as effective heterogeneous nuclei. Mineral substrate [11], silanised mica surface [12], lipid bilayers deposited on glass cover slips (for membrane protein crystallisation) [13,14], polymeric film containing poly-l-lysine or poly-l-aspartate ionisable groups [15], and a modified surface with a varying roughness [16,17] have all been proven to be useful for promoting protein crystallisation. These findings demonstrated that heterogeneous nucleation can effectively occur on large surfaces. If the surfaces can serve directly as the crystallisation plate or glass cover slip, the additional procedure of adding the nuclei can be avoided, and the heterogeneous nucleation can be more easily applied in high-throughput protein crystallisation.

Based on this concept, the surface modification of the crystallisation plate for sitting-drop crystallisation or modification of the glass cover slip for hanging-drop crystallisation can be very useful and beneficial. There are many methods for surface modification, among which the deposition of a self-assembled monolayer (SAM) is unique. A SAM is formed spontaneously by the absorption of an organic molecule onto a surface to generate a lipid-like film [18]. The properties of the SAM depend on the type of material such that it is possible to prepare a SAM that meets a specific functional requirement. Examples include the capabilities of protein absorption [19–21], platelet adhesion [22], and leukocyte adhesion [23].

The SAMs are films with a high degree of ordered structure, which may be helpful for protein crystallisation because the latter is also a process of packing protein molecules in an ordered structure. David and Pham *et al.* have investigated the nucleation of protein crystals on a methyl-modified SAM (gold-coated glass cover slip) and found that the nucleation was facilitated and accelerated on such a surface [24,25]. This result indicated that SAMs may be potentially useful in high-throughput protein crystallisation. It should be more applicable in practical crystallisation if the SAMs are deposited directly on cover glass so that there will be no gold layer to block the optical path for crystal inspection.

In this paper, we report a method to prepare SAMs modified with functional groups (including methyl, sulfydryl and amino groups, which commonly exist in proteins) directly on the surface of the glass cover slip. To examine the effect of these SAMs on protein crystallisation, we performed crystallisation screening and reproducibility tests. The modified surfaces were also characterised to understand the possible mechanisms for their observations in the experiment.

## 2. Results

### 2.1. Characterisation of the Modified SAMs

**Figure 1:**
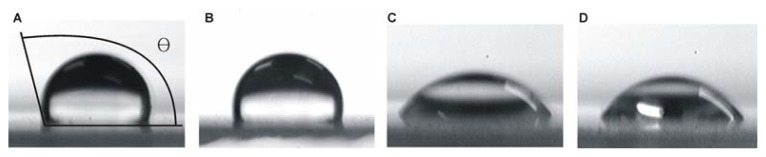
Measurement of the contact angles of the silanised (**A**); methyl-modified (**B**); sulfydryl-modified (**C**); and amino-modified (**D**) cover slips. The contact angle θ is shown in (A).

The SAMs that formed on the cover slips were characterised by determining the contact angles, which were consistent with previously published values (Table 1). Figure 1 shows the images for the contact angles of the silanised slip and the modified SAMs slip, which demonstrated an obvious decrease in the contact angle, especially for the amino- and sulfydryl-modified SAMs. The contact angle decreased from 111.0° for the silanised cover slip to 104.0°, 41.9° and 49.1° for the methyl-, amino- and sulfydryl-modified cover slips, respectively. The silanised cover slip demonstrated the most hydrophobic surface followed by the methyl-, sulfydryl- and amino-modified cover slips.

**Figure 2:**
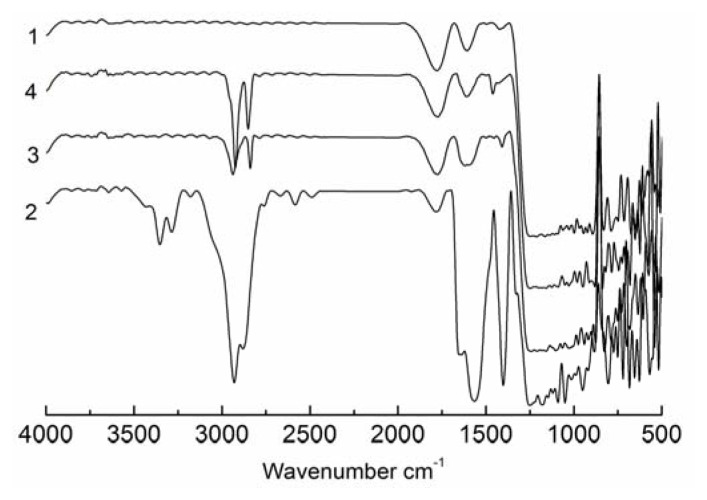
FTIR analysis. The FTIR spectra for the untreated cover slip and the amino-, sulfydryl- and methyl-modified SAMs are shown in 1, 2, 3 and 4, respectively.

The chemical composition of the SAMs were analysed using FTIR and XPS. The FTIR spectra are shown in Figure 2. The region between 3000 and 2800 cm^−1^ in the spectrum for the untreated glass cover slip did not contain any peaks, which indicated the absence of hydrocarbons on this surface. For the amino-modified cover slip, peaks were observed at 1553 cm^−1^ and 1649 cm^−1^, which represent the deformation vibration of NH_2_, and the peaks at 3365 cm^−1^ and 3284 cm^−1^ are attributed to the stretching vibration of N–H in NH_2_. For the sulfydryl-modified cover slip, the peaks at 2938 cm^−1^ and 2843 cm^−1^ represent the stretching vibration of S–H. For the methyl-modified cover slip, the peaks at 2918 cm^−1^ and 2849 cm^−1^ are attributed to the stretching vibration of C–H in CH_2_ and CH_3_.

**Figure 3:**
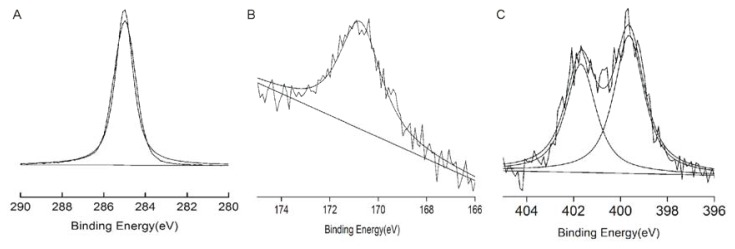
High-resolution XPS C1s (**A**), S2p (**B**) and N1s (**C**) spectra of (**A**) methyl-, (**B**) sulfydryl- and (**C**) amino-modified SAMs with peak fitting. In the N1s spectra, the first peak at a binding energy of 402 eV represents the positively charged element.

Figure 3 shows typical high resolution XPS C1s spectra for the monolayers. In Figure 3A, a carbon species (284.8 eV) was present in the spectrum of the methyl-modified cover slip. In Figure 3B, the sulphur peak was observed at a binding energy of 171 eV. In Figure 3C, two nitrogen peaks were observed at 402 eV (positively charged) and 399 eV (no charge); thus, the surface of the amino-modified cover slip was partially positively charged. The details of XPS analysis of the SAM surfaces are presented in Table 1 and indicate that these results lie within the expected range for each SAM-modified surface. The results of both XPS and FTIR suggested that these groups were well modified on the surface of the cover slips.

**Figure 4:**
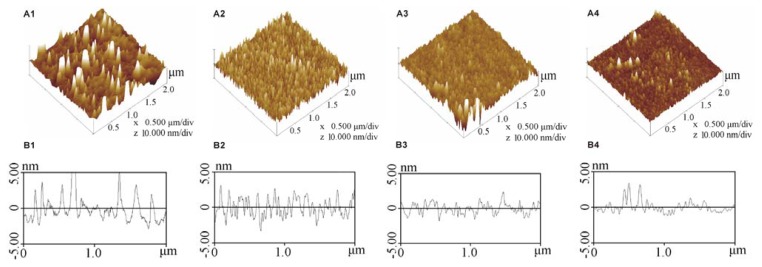
AFM 3D micrograph (**A1**–**A4**) and profile diagram (**B1**–**B4**) of the silanised cover slip and SAMs. The images from 1 to 4 represent silanised, methyl-, sulfydryl- and amino-modified cover slips, respectively.

Figure 4 shows the morphology of the silanised, methyl-, sulfydryl- and amino-modified cover slips, and their average roughness was 2.271 nm, 1.486 nm, 1.174 nm and 0.978 nm, respectively, which indicated that the surface of the silanised cover slip slightly rougher than that of the modified SAMs, and the structure of the modified SAMs was more well ordered than on the silanised cover slip (Figure 4).

### 2.2. Effect of the Modified SAM Cover Slips on the Reproducibility of Protein Crystallisation

**Figure 5:**
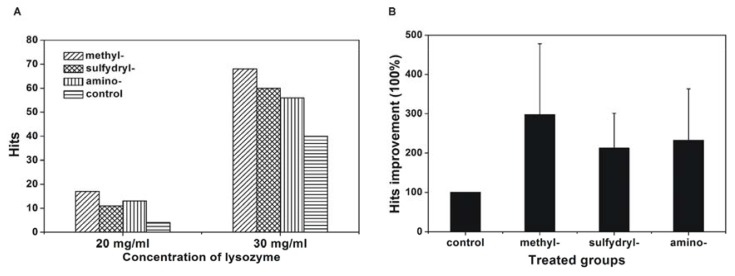
Crystallisation hits (**A**) and hits improvement (**B**) using modified SAMs. The concentration of lysozyme was 20 mg/mL and 30 mg/mL before mixing, and the concentration of NaCl was 60 mg/mL before mixing.

Low reproducibility of protein crystallisation has always been an obstacle. Because reproducibility samples a large number of repeated crystallisation experiments, it can be used to evaluate protein crystallisation. Therefore, the effect of the modified SAMs on the reproducibility of protein crystallisation was investigated here. Lysozyme was chosen as a model protein for the reproducibility study at two concentrations, 20 mg/mL and 30 mg/mL before mixing, using the silanised cover slip as the control. The results are shown in Figure 5 and indicate that the reproducibility of crystallisation was enhanced on these modified surfaces, and this enhancement was more significant at a low concentration of lysozyme. At a lysozyme concentration of 20 mg/mL, the crystallisation reproducibility improved 4.25 times on the methyl-modified cover slip, 2.75 times on the sulfydryl-modified cover slip, and 3.25 times on the amino-modified cover slip. To determine the significance of the improvement in the number of hits using the different modified SAMs compared with the silanised cover slip, a one sample *t*-test was applied. The number of hits using the silanised cover slip was set as 1. The percentage of hits improvement was calculated by dividing the number of hits using the modified SAMs by that using the silanised cover slip. The means percentage of hits improvement of all proteins in this investigation under each modified SAMs was calculated. Percentage of hits improvement using each modified SAMs was compared with that of silanised cover slip, it indicted that *p* value was less than 0.05, so the difference of hits improvement between these groups was statistically significant, and this improvement was most significant for the methyl-modified cover slip (hits improvement of 297.5% ± 180.3%) followed by 212.5% ± 88.4% improvement for the sulfydryl-modified cover slip and 232.5% ± 130.8% improvement for the amino-modified cover slip. All of these results indicated that the crystallisation was facilitated on the modified SAMs, and the highest number of hits was obtained using the methyl-modified cover slip followed by the sulfydryl- and amino-modified cover slip.

### 2.3. Effect of Modified SAMs on Protein Crystallisation Screening

To further determine the effect of the modified SAMs on protein crystallisation, a screening experiment was performed. Eleven commercial proteins were chosen, and it was found that protein crystallisation was promoted on the modified SAMs, especially for glucose isomerase and ribonuclease A type XII. For glucose isomerase, the percentage of hits increased from 27.1% for the control to 76.0% for the methyl-modified cover slip, 67.7% for the sulfydryl-modified cover slip, and 76.0% for the amino-modified cover slip. For ribonuclease A type XII, only one hit was obtained for the control, and four hits were obtained using the methyl-modified cover slip, six using the sulfydryl-modified cover slip, and four using the amino-modified cover slip.

**Figure 6:**
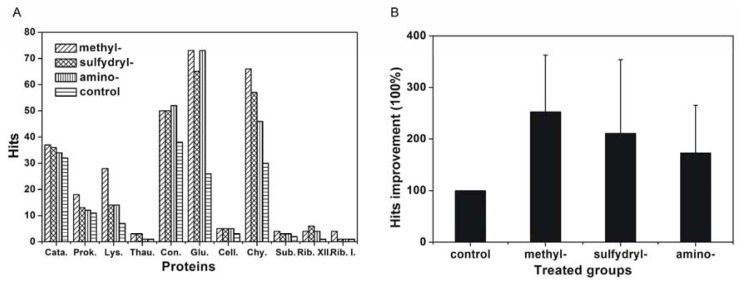
Crystallisation screening hits (**A**) and hits improvement (**B**) using SAM-modified cover slips. Cata.: catalase; Prok.: Proteinase K; Lys.: lysozyme; Thau.: thaumatin; Con.: concanavalin A type VI; Glu.: glucose isomerase; Cell.: cellulase; Chy.: α-chymotrypinoyen A type II; Sub.: subtilisin A type VII; Rib. XII: ribonuclease A type XII; and Rib. I: ribonuclease A type I.

The success rate of crystallisation screening utilising the modified SAMs was further analysed by one sample *t*-test. The improvement in hits was defined in an identical manner as in the crystallisation reproducibility study. The results are presented in Figure 6. The P values were less than 0.05, so the improvement in the number of hits was significantly different between modified SAMs compared with the silanised cover slip. The average percentage of hits improvement was 252.6% ± 109.9% for the methyl-modified cover slip, 210.8% ± 142.8% for the sulfydryl-modified cover slip, and 173.0% ± 92.38% for the amino-modified cover slip. Therefore, the hits improvement for the success rate of crystallisation screening using the modified SAMs was significant, and hits improvement was most significant for the methyl-modified cover slip followed by the sulfydryl- and amino-modified cover slip.

### 2.4. Effect of Modified SAMs on the Number of Crystals

**Figure 7:**
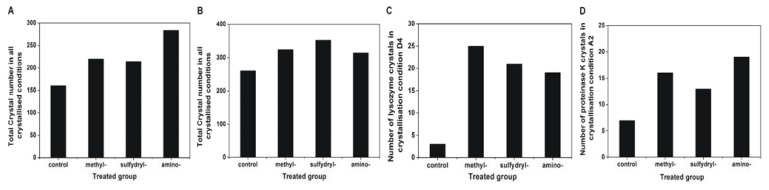
Number of protein crystals obtained with different modified SAMs. The overall number of crystals of lysozyme (**A**) and proteinase K (**B**) in all crystallised conditions. The number of lysozyme crystals in the crystallisation condition D4 (**C**), and the number of proteinase K crystals in the crystallisation condition A2 (**D**). The screening kit Index™ from Hampton Research was used.

Because the number of crystals can indicate the effect of the different SAMs on the protein nucleation process, the number of crystals grown on the various modified SAMs was determined. Lysozyme and proteinase K were chosen as the model proteins for this investigation. The number of crystals increased on the modified SAMs for both proteins, which was consistent with the results of the crystallisation reproducibility and screening (Figure 7). To further verify that the effect of these modified SAMs on the number of crystals was not caused by the number of crystallisation conditions but by the different SAMs, two crystallisation conditions in which protein crystals appeared on all of the modified SAMs were selected to compare the number of obtained crystals (D4 for lysozyme: 0.1 M citric acid, pH 3.5, and 25% *w*/*v* polyethylene glycol 3350; A2 for proteinase K: 0.1 M sodium acetate trihydrate, pH 4.5, and 2.0 M ammonium sulphate). The results further confirmed that the number of obtained crystals increased on the modified SAMs.

**Figure 8:**
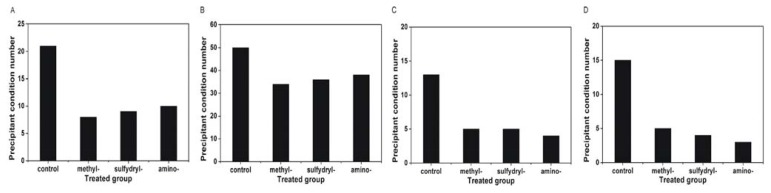
The number of conditions producing precipitate using different modified SAMs. The overall number of conditions producing precipitate for concanavalin A type VI (**A**), catalase (**B**), α-chymotrypinoyen A type II (**C**) and glucose isomerase (**D**) from the crystallisation screening using the Index™ kit from Hampton Research.

**Figure 9:**
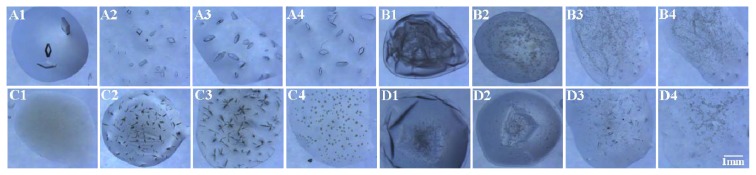
Images of the droplets on SAMs of different groups and the control. Images of crystals of lysozyme in the crystallisation condition D4 (**A1**–**A4**), concanavalin A in the crystallisation condition D10 (**B1**–**B4**), catalase in the crystallisation condition D1 (**C1**–**C4**), and α-chymotrypinoyen A type II (**D1**–**D4**) in the crystallisation condition H11. The Index™ screening kit from Hampton Research was used. The images of the droplets from 1 to 4 represent the control, methyl-, sulfydryl- and amino-modified cover slips, respectively.

The appearance of precipitate in the crystallisation trials indicates a decrease in the protein concentration. Thus, the success rate of protein crystallisation decreases for conditions that produce precipitate. Therefore, the number of conditions producing precipitate was compared with the control (Figure 8). Concanavalin A type VI, catalase, α-chymotrypinoyen A type II and glucose isomerase were selected as model proteins, as the precipitate was more easily formed during crystallisation for these proteins. The results indicated that the number of conditions producing precipitate decreased using the modified SAMs. It was found that the protein tends to precipitate on the silanised cover slip compared with the modified SAMs, which may explain the high crystallisation success rate using the modified SAMs. The images of the crystals obtained using the modified SAMs and the control are shown in Figure 9 (lysozyme at condition D4:0.1 M citric acid, pH 3.5, and 25% *w*/*v* polyethylene glycol 3350; concanavalin A at condition D10:0.1 M BIS-TRIS, pH 6.5, and 20% *w*/*v* polyethylene glycol monomethyl ether 5000; catalase at conditions D1:25% *w*/*v* polyethylene glycol 1500; and α-chymotrypinoyen A type II at conditions H11:0.1 M potassium thiocyanate and 30% *w*/*v* polyethylene glycol monomethyl ether 2000). The results indicated that more crystals and less precipitate appeared on the modified SAMs compared with the control.

### 2.5. Crystal Orientation on the Amino-Modified Cover Slip

**Figure 10:**
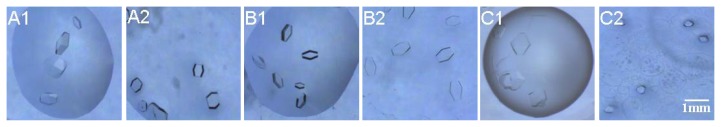
The effect of the amino-modified cover slip on the orientation of lysozyme (**A** and **B**) and glucose isomerase (**C**). Crystals were obtained using the silanised cover slip (**A1**, **B1** and **C1**) and amino-modified cover slip (**A2**, **B2** and **C2**). The concentration of lysozyme in A1, A2, B1 and B2 was 20 mg/mL before mixing, and the crystallisation reagent in A1 and A2 was 60 mg/mL NaCl before mixing; the crystallisation reagent in B1 and B2 was condition D5 from the Index™ screening kit from Hampton Research. The concentration of glucose isomerase in C1 and C2 was 7 mg/mL before mixing, and the crystallisation reagent was condition F3 from the Index™ screening kit from Hampton Research. The condition D5 contains 0.1 M sodium acetate trihydrate, pH 4.5, and 25% *w*/*v* polyethylene glycol 3350; the condition F3 contains 5% *v*/*v* Tacsimate, pH 7.0, 0.1 M HEPES, pH 7.0, and 10% *w*/*v* polyethylene glycol monomethyl ether 5000.

The surface of the amino-modified cover slip was positively charged, which was confirmed by FTIR. If an attractive force was present between the charged protein molecule and the SAM surface, the orientation of the crystals would be detected. Two proteins were selected for this study, lysozyme (pI = 11.3, which was positively charged in the chosen crystallisation condition) and glucose isomerase (pI = 3.0, which was negatively charged). The results indicated that both proteins demonstrated an orientation (Figure 10).

The positive charge on the amino-modified cover slip should attract a negatively charged protein and thus increase protein adsorption and enhance protein crystallisation. To further confirm whether crystallisation can be promoted by the attractive force between the protein and the SAM surface, the correlation between hits and protein charge was analysed, and the results are summarised in Table 2. The SAM demonstrating the methyl-modified SAM (the highest number of hits) in this study was utilised as a control, and the ratio of hits between the methyl- and amino-modified cover slips was calculated. The results indicated that the number of hits using the amino-modified cover slip for all positively charged proteins was lower than that using the methyl-modified cover slip (except ribonuclease A type XII and subtilisin A), while this difference in the number of hits decreased for all of the negatively charged proteins.

## 3. Discussions

Protein nucleation is the initial step of crystallisation and only occurs when the supersaturation is sufficiently high to overcome the nucleation barrier [26]. The fluctuation in the concentration (from under to super saturation) and structure (low to high order) is necessary for nucleation [27]. In detail, the fluctuation in the concentration facilitates the formation of a local high concentration area, which is beneficial to overcome this nucleation barrier [28]. The fluctuation in structure is helpful to form well-ordered clusters, which are more easily packed during crystallisation and promote protein nucleation [5]. In this study, protein crystallisation was facilitated on groups modified SAMs, which can be explained by an increase in the fluctuation in concentration (increasing local supersaturation) and structure (which is beneficial for crystal orientation) such that protein crystallisation was promoted.

### 3.1. Increasing Opportunities of Heterogeneous Nucleation on Modified SAMs

The energy barrier of nucleation is reduced at the liquid-solid interface, which has always been utilised for heterogeneous nucleation; thus, protein crystals have always been found at the regions of the droplet that contact the crystallisation apparatus [29]. A large contact surface area is beneficial to increase the opportunities of heterogeneous nucleation [30].

The contact area of crystallisation droplets on the various modified SAMs was investigated. Based on the droplet volume and contact angle, the ratio of the liquid-solid interface area between each modified SAM and the silanised surface (control) was calculated: *S_methyl_/S_control_* = 1.2, *S_amino_/S_control_* = 3.4, and *S_sulfydryl_/S_control_* = 4.3. These results indicated that the contact area increased on the modified SAMs. The larger liquid-solid interface area of the modified SAMs provided more opportunities for heterogeneous nucleation, which facilitated nucleation.

### 3.2. Increasing the Fluctuation in Concentration on the Modified SAMs

Heterogeneous nucleation occurred more easily on the surface of the modified SAMs. A local high concentration area was necessary for growing these nuclei; otherwise, these existing nuclei would dissolve again [5], which makes the fluctuation in concentration necessary.

AFM indicated that the morphology of the SAMs was more ordered than silanised cover slip. Protein crystallisation is a process of packing molecules in a well-ordered way [31]. Therefore, these molecules were more easily packed on the well-ordered surface of the SAMs to form a crystal rather than precipitate, which was confirmed by the results that precipitate was more easily formed on the silanised cover slip compared to the well-ordered modified SAMs. Thus, the protein concentration decreased on the silanised cover slip, and the protein concentration was higher on the modified SAMs compared with that on the silanised cover slip.

The evaporation rate increases with a larger surface area of the crystallisation droplets, which concentrates the protein more easily and is beneficial for nucleation. Therefore, the ratio of the surface areas of the droplets on the modified SAMs and the silanised cover slip (control) was calculated and are *S′_methyl_/S′_control_* = 1, *S′_amino_/S′_control_* = 1.3, and *S′_sulfydryl_/S′_control_* = 1.68. These results indicated that the surface area of the droplets increased on the modified SAMs, thereby concentrating the crystallisation droplet more easily on the modified SAMs and promoting protein crystallisation.

**Figure 11:**
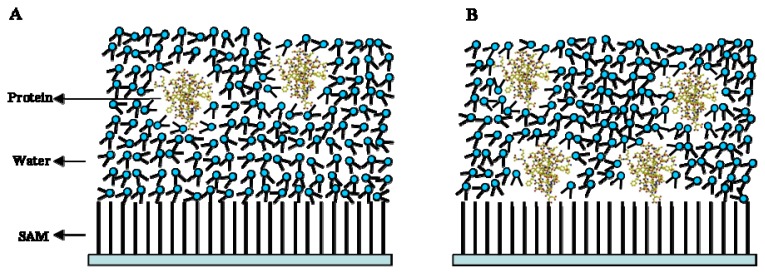
The model of protein adsorption on the (**A**) amino- or sulfydryl- and (**B**) methyl-modified SAMs. For the methyl-modified cover slip, the protein can easily interact with the modified SAM [32].

Except the evaporation, protein adsorption is also important for protein crystallisation. We observed that the nucleation did occur at air-water interfaces due to droplet evaporation. However, most crystals were found to adhere to the surface-water interfaces, which indicated that nucleation mainly occurred on the surface-water interfaces, so protein adsorption on the surface of the modified SAMs is another important factor for fluctuation in the protein concentration. Both protein and water molecules can interact with the contact surface through hydrogen bonds, and they competitively interacted within the limited area of the contact surface. In this case, water molecules more easily interact with the contact surface due to its smaller size. Therefore, a water barrier was always formed on the contact surface, which disturbs the interaction between protein molecules and the contact surface, thus limiting protein absorption. For amino-modified, sulfydryl-modified and silanised cover slip, a hydrogen-bonding acceptor was present on these surfaces, and water molecules interacted with these modified SAMs through hydrogen bonds forming a water barrier that disturbed protein adsorption. In contrast, the methyl-modified cover slip does not contain a hydrogen-bonding acceptor on the methyl group. Therefore, water molecules do not adhere on the contact surface, and the formation of the water barrier was disturbed. Simultaneously, the protein molecules interact with the methyl-modified cover slip through van der Waals forces, thereby increasing the protein concentration and simultaneously producing a fluctuation in the concentration, it had been well clarified using molecular simulation [32] (Figure 11). Circular dichroism (CD) analysis has confirmed that the protein concentration increases on the surface of the modified SAMs, which occurred more significantly on the methyl-modified cover slip [33,34]. For the sulfydryl-modified cover slip, disulphide bonds commonly exist in most proteins; therefore, the sulfydryl-modified cover slip could interact with disulphide bonds in proteins [35], thereby inducing an increase in the protein concentration on the surface of the SAM.

### 3.3. Increase of the Fluctuation in Structure on Modified SAMs

The fluctuation in structure is another determining factor for crystallisation, and it increases with the well-ordered packing of molecules. The amino-modified cover slip was positively charged, and this electrical potential aided in the orientation of the protein molecules on these cover slips, enhancing the ordered packing of the protein molecules, thereby increasing the fluctuation in structure on the SAM, and promotes protein crystallisation.

## 4. Experimental Section

### 4.1. Materials

Eleven commercial proteins (as listed in Table 3) were used without further purification. Sodium chloride was purchased from the Chemical Reagent Co. Ltd. (Beijing, China). Acetone, sulphuric acid, acetic acid and sodium acetate were obtained from Beijing Chemical Factory (Beijing, China). HEPES sodium was obtained from Amresco (Solon, OH, USA). Dimethyldichlorosilane, hexadecyltrimethoxysilane, γ-mercaptopropyltrimethoxysilane and γ-aminopropyltrimethoxysilane were obtained from Sigma-Aldrich Co. (St. Louis, MO, USA). The crystallisation screening kit Index™ was obtained from Hampton Research Co. (Aliso viejo, CA, USA). Cover slips were purchased from Shanghai Electric International Economic & Trading Co. (Shanghai, China) (size: 18 mm × 18 mm, thickness: 0.13 mm–0.17 mm).

### 4.2. Experiments

#### 4.2.1. Preparation of Modified SAMs and Silanised Cover Slips

**Figure 12:**
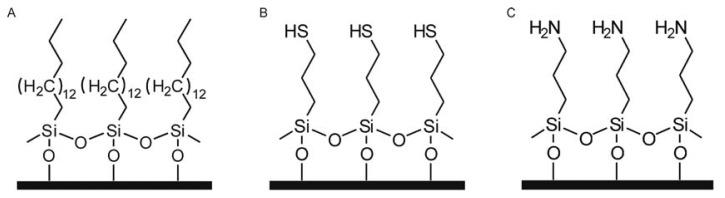
Schematic illustration of the (**A**) methyl-; (**B**) sulfydryl-; and (**C**) amino-modified cover slips.

To prepare suitable substrates for the self-assembling monolayers, the cover slips were sonicated three times in 95% ethanol, rinsed with deionised water, immersed in a solution (3:1 *v*/*v* H_2_SO_4_/H_2_O_2_) for 24 h followed by washing with deionised water until the pH was neutral. Finally, the cover slips were dried with N_2_ and were ready for use. The above treated cover slips were soaked in silane solution (hexadecyltrimethoxysilane for methyl-modified SAMs, γ-mercaptopropyltrimethoxysilane for sulfydryl-modified SAMs and γ-aminopropyltrimethoxysilane for amino-modified SAMs) diluted with acetone solution to a final concentration of 0.03%. A small amount of acetic acid was added to catalyse the hydrolysis of Si–O–CH_3_. After 2 h, the cover slips were rinsed with acetone to remove excess silane solution. The modified SAMs formed after drying the acetone-rinsed cover slips. Figure 12 schematically illustrates the methyl-, sulfydryl-, and amino-modified SAMs.

As silanised cover slips are commonly used in the hanging-drop crystallisation method, it was chosen as a control in this experiment. The procedure for preparing silanised cover slips was as follows: the cover slips were sonicated three times in 95% ethanol, rinsed with deionised water, immersed in dimethyldichlorosilane for 30 min, and rinsed with deionised water. Once dry, the silanised films formed on the cover slips.

#### 4.2.2. Analysis of the SAMs

The modified cover slips were characterised by contact angle goniometry, Fourier transform infrared spectroscopy (FTIR) and X-ray photoelectron spectroscopy (XPS).

The contact angle was measured using a JC2000C Contact Angle Meter from Powereach Co. (Shanghai, China). A drop of 5 μL of deionised water was deposited on the cover slip, and 2–6 s were required for the measurement. The measurement temperature was 293 ± 2 K. Each measurement was repeated five times.

The SAMs were analysed by FTIR spectroscopy (TENSOR 27 FTIR spectrometer, Bruker, Germany).

XPS was used to determine the elemental composition of the monolayer. After the formation of the SAMs, the samples were analysed by K-alpha X-ray photoelectron spectroscopy (Thermo Fisher Scientific, Pittsburgh, PA, USA) using monochromatic A1 Kα radiation (150 W, 15 kV, 1486.6 eV). The vacuum of the spectrometer was set at 1.33 × 10^−5^ Pa during the measurement. The elemental composition was determined from the peak areas in the spectra, and the analysis was performed using Avantage v4.51 (Thermo Fisher Scientific, Pittsburgh, PA, USA).

The surface morphologies of the SAMs were studied using atomic force microscopy (Nanoscope IIIa, Digital Instruments, New York, NY, USA). The temperature was 295 K; the relative humidity was 39.5%; and the scanning was performed in tapping mode.

#### 4.2.3. Crystallisation Experiments

Protein crystallisation experiments were performed in two parts: screening and reproducibility studies. The hanging-drop method was utilised in both studies.

Screening was used to examine the effect of the SAMs on protein crystallisation screening. Protein solutions were prepared by dissolving the proteins in their corresponding buffers (as in Table 3). These solutions were filtered by filters with a pore size of 0.22 μm (SLGV033RS, Millipore, Billerica, MA, USA). The crystallisation trials were set up by mixing the protein solution with the crystallisation reagents from the Index™ screening kit (Hampton Research, Aliso viejo, CA, USA) at a volume ratio of 1 μL:1 μL. The volume of each reservoir solution was 80 μL. The crystallisation plates were prepared by filling each well of the 40-well plates (Keyu, Jiangsu, China; each well had a diameter of 7 mm and a depth of 10 mm). After setting up the crystallisation trials, the crystallisation plates were placed into a sealed chamber (inner dimensions: 28 cm × 23 cm × 11 cm). The chamber was connected to a programmable refrigerated circulator (PolyScience 9712 refrigerated circulator, PolyScience Inc., Niles, IL, USA) such that the temperature inside the chamber could be accurately controlled within ±0.1 K. In this study, the temperature for the screening study was 293 K, and the incubation time was 96 h. After incubation, the images of the crystallisation droplets were captured by an automated crystal image reader (XtalFinder XtalQuest Inc., Beijing, China) equipped with a UV light source (CRYSTALIGHT™ 100 UV source, Molecular Dimensions, Altamonte Springs, FL, USA) [36]. By comparing the crystallisation hits (here, “hits” are defined as the number of crystallisation conditions that yield observable protein crystals under the microscope in this experiment), we could determine the effects of different SAMs on protein crystallisation screening.

Similarly, the effect of the SAMs on the reproducibility of crystallisation was examined. Because a reproducibility study can sample a large number of repeated crystallisation experiments, it can be applied to verify an effect that is identified in the screening study. The experimental procedures of the reproducibility study are as follows: lysozyme was selected as a model protein and was dissolved in 0.1 M sodium acetate, pH 4.6. Two different concentrations (the concentrations before mixing were 20 mg/mL and 30 mg/mL) of lysozyme were used. The concentration of NaCl was 60 mg/mL before mixing. The crystallisation on each SAM was repeated 40 times. The temperature was controlled at 20 °C, and the incubation time was 96 h. The remaining procedures are identical to those described in the screening study.

## 5. Conclusions

Heterogeneous nucleation is an available method to improve protein crystallisation and is a promising approach to overcome the barrier of nucleation. The surface of the crystallisation apparatus can serve as the easiest source of heterogeneous nuclei. In this study, the cover slip was modified by SAMs with different functional groups, including methyl, sulfydryl and amino, and the effects of these modified SAMs on protein crystallisation screening and reproducibility were investigated. Protein crystallisation was significantly promoted on these modified SAM cover slips. The possible mechanisms regarding increased opportunities for heterogeneous nucleation, protein concentration and fluctuation in structure were discussed. It was proposed that these modified SAMs could be used as a promising and easy tool to enhance protein crystallisation.

## Figures and Tables

**Table 1:** Contact angle and elemental composition of the self-assembled monolayer (SAM) surface.

Treatment	CA a	Si (%)	O (%)	C (%)	N (%)	S (%)	Cl (%)
untreated	18.4	61.9	38.1	-	-	-	-
silanised	111.0	51.0	34.9	14.1	-	-	0.1
methyl-modified	104.0	45.1	33.2	21.8	-	-	-
sulfydryl-modified	49.1	58.0	35.2	6.8	-	0.1	-
amino-modified	41.9	57.7	34.7	6.4	1.2	-	-

a CA represents the contact angle; - indicates negligible.

**Table 2:** Protein charge distribution on the amino-modified cover slip.

Protein	pI	Protein charge	Hits		Ratio of hits between methyl- and amino-modified SAMs

			methyl-modified SAMs	amino-modified SAMs	
lysozyme	11.3	positive	28	14	0.50
subtilisin A type VII	9.4	positive	4	3	0.75
thaumatin	12.0	positive	3	1	0.33
ribonuclease A type I	9.6	positive	4	1	0.25
ribonuclease A type XII	9.6	positive	4	4	1.00
α-chymotrypsinogen A type II	9.0	positive	66	30	0.70
proteinase K	9.0	positive	18	12	0.67
catalase	5.0	negative	37	34	0.92
concanavalin A type VI	5.5	negative	50	52	1.04
glucose Isomerase	3.0	negative	73	73	1.00
cellulase	4.8	negative	5	5	1.00

**Table 3:** Proteins and their corresponding buffers used in the investigation.

No.	Protein	Catalogue No.	Supplier	Buffer
1	lysozyme	E05801	Seikagaku	100 mM sodium acetate, pH 4.6
2	catalase	C40	Sigma-Aldrich	25 mM HEPES sodium, pH 7.0
3	subtilisin A type VII	P5380	Sigma-Aldrich	25 mM HEPES sodium, pH 7.0
4	thaumatin	T7638	Sigma-Aldrich	25 mM HEPES sodium, pH 7.0
5	concanavalin A type VI	L7647	Sigma-Aldrich	25 mM HEPES sodium, pH 7.0
6	ribonuclease A type I	R4875	Sigma-Aldrich	25 mM HEPES sodium, pH 7.0
7	ribonuclease A type XII	R5500	Sigma-Aldrich	25 mM HEPES sodium, pH 7.0
8	α-chymotrypinoyen A type II	C4879	Sigma-Aldrich	25 mM HEPES sodium, pH 7.0
9	proteinase K	P6556	Sigma-Aldrich	25 mM HEPES sodium, pH 7.0
10	glucose isomerase	HR7-100	Hampton Research	25 mM HEPES sodium, pH 7.0
11	cellulase	C0615	Sigma-Aldrich	25 mM HEPES sodium, pH 7.0
